# Development of emergency nursing care competency scale for school nurses

**DOI:** 10.1186/s12912-021-00580-9

**Published:** 2021-04-14

**Authors:** Jaehee Yoon

**Affiliations:** Wolchon Elementary School, 132, Mokdongjungang-ro, Yangcheon-gu, Seoul, 07980 South Korea

**Keywords:** Emergency nursing care, School nursing, Competency, Scale, Instrument

## Abstract

**Background:**

School nurses perform vital student emergency services at school, and assessing their emergency nursing care competency is critical to the safety and quality of care students receive. The purpose of the study was to develop a scale for measuring school nurses’ competency.

**Methods:**

This was an instrument development and validation study. It was conducted according to the revised DeVellis scale development process coupled with the application of the International Council of Nurses’ Nursing Care Continuum Competencies Framework. Eight experts specializing in school health and emergency care evaluated the content validity, while 386 school nurses evaluated the scale. The validity evaluation comprised factor analysis, discriminative validity analysis according to differences in school nurse experience, and criterion validity analysis. Scale internal consistency was analyzed using Cronbach’s α value.

**Results:**

The final scale comprises a self-reported 5-point Likert scale with 30 items based on three factors and three sub-factors. Both the convergent validity of the items by factor and the discriminative validity were both confirmed. The criterion validity was also found to be positively correlated with the Triage Competency Scale.

**Conclusion:**

The scale may be used to identify factors influencing school nurses’ competency in emergency nursing care and contribute to research in competency-based education programs.

**Supplementary Information:**

The online version contains supplementary material available at 10.1186/s12912-021-00580-9.

Student medical emergencies at schools precipitated by accidents and health problems are common occurrences these days given the considerable amount of time students spend at school for educational activities [1, 2]. For example, the incidence of sudden cardiac arrest per 100,000 students ranged from 0.17 to 4.4 in the United States [3] and reached 0.4 in Japan [4]. In South Korea, the number of school accidents increased from 86,468 in 2011 to 122,570 in 2018 [5], while the number of students diagnosed with rare or chronic illnesses grew from 1626 in 2017 to 1758 in 2018 [6], making student medical emergencies at school more likely than ever [7–10].

School nurses represent the only medical professionals available to provide emergency care in schools [11, 12], making their Emergency Nursing Care Competency (ENCC) a vital component of the school emergency services. The ENCC is also a critical factor in school emergency response given the primary role they play in planning emergency care and training school staff [10, 13]. In particular, ENCC is essential for a safe and positive student care outcome because school nurses make student care decisions largely on their own [14, 15].

Competency scales, which can be used to effectively assess nursing competency, contribute to maintaining and improving competency [16]. In fact, Resha [17] contended that competency evaluation constitutes a critical aspect of maintaining competency and establishing improvement methods. Competency assessment can also be used to improve the existing practices and promote continuing education [16, 18, 19]. Previous studies [20–22] on the factors affecting nurse competency might help identify effective ways to improve competency, and competency scales have been used in such research. In addition, many studies [23, 24] have shown that competency scales could be used to develop competency-based educational programs and test their effectiveness [18, 25].

To date, however, no scale capable of measuring school nurses’ ENCC has been developed. There are previous studies [26, 27] on the overall competencies of school nurses, but they invariably suffered from limitations regarding identifying the content and level of the ENCC. Given these challenges, the current study aimed to develop and validate a scale to measure school nurses’ ENCC.

## Methods

### Procedures

**Fig. 1:**
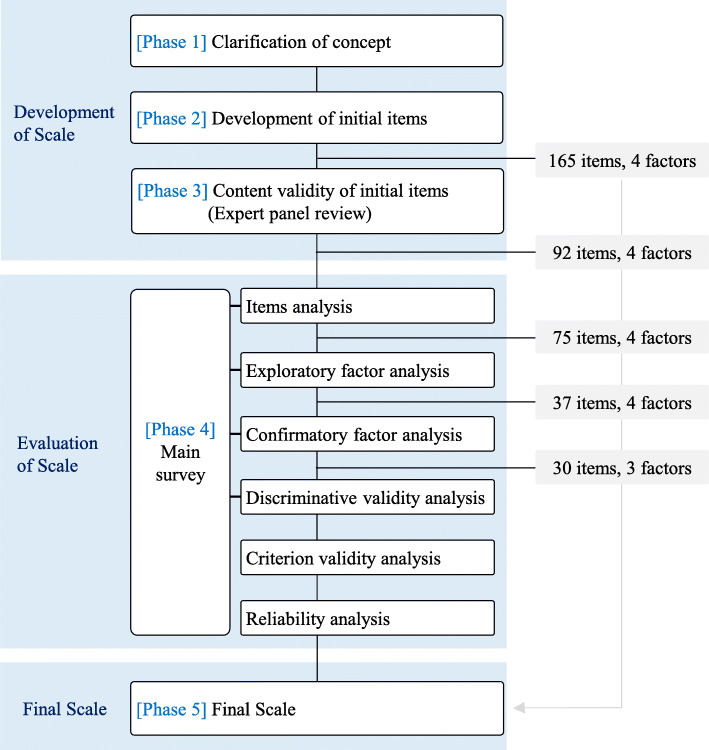
Scale development process and change based on the revised DeVellis scale development process [28]

This was an instrument development and validation study. The study was conducted according to the revised DeVellis scale development process [28]. Since this process was primarily theoretical, it needed to be modified for data collection and analysis. The revised process was classified into five phases, with the scale being completed by changing the number of items and factors during each phase (Fig. 1).

### Scale development

#### Phase 1: clarification of concept

The concept was analyzed and developed in detail using the Nursing Care Continuum Competencies Framework (NCCCF), consisting of 3 factors, competencies, and behavioral indicators [29]. Some of the processes used by Elo and Kyngas [30] for qualitative content analysis, such as preparation and organization, were applied.

In the preparation phase of the content analysis, we reviewed the dictionary of terms and conceptual analysis studies [31–33] for each competency listed in the NCCCF [29] to understand their meaning. We also added behavioral indicators or competencies to the NCCCF [29] by reviewing previous studies [10, 26, 27, 34–36]. We selected previous studies were searched using the following search criteria:

1. Date: from 2007 to 2017
2. Search terms
3. [(emergenc* OR crisis OR critical OR Hotlines OR nurs* OR service* OR responders OR care OR acute care OR patient care, prehospital OR trauma nursing OR emergency aid OR urgent medical aid OR first-aid) AND (competenc*): ti]
4. Databases: KISS, KISTI, Dbpia, and EMBAS
5. Exclusion criteria: No relevant to study subject, full text, or not written in Korean or English
6. Hand search: The Journal of School Nursing, the Journal of School Health, and the Journal of Korean Society of School Health, the references to the cited studies

The identified content from the preparation stage was coded in words or phrases after multiple readings in the organizational phase. The coded content was then collected and categorized into themes, which were written as school nurse’s competency and behavioral indicators with due consideration given to the characteristics of the school environment.

#### Phase 2: development of initial items

Initial items were developed on the basis of the behavioral indicators derived during Phase 1. Data from the School Safety Insurance Federation from 2015 to 2016 [5] were analyzed to identify the type and frequency of school emergencies.

#### Phase 3: content validity of initial items (expert panel review)

We formed an expert panel comprised of nine members: one emergency nurse, one emergency head nurse, one professor of nursing with school nurse experience, one professor of nursing with emergency nurse experience, one professor of community nursing, one supervisor for school health, and three school nurses enrolled in a doctoral nursing program.

A total of 18 competencies, 65 behavioral indicators, and 165 items were assessed for their content validity. The nine modified items from the Triage Competency Scale (TCS) for emergency nurses that had not fit the school context were also reviewed [35].

The Content Validity Index for Item (CVI-I) was computed using the number of the experts’ ratings of 3 or 4 on a 4-point scale (1 = *not relevant*, 2 = *somewhat relevant*, 3 = *quite relevant*, 4 = *highly relevant*) given to each item, divided by the total number of experts [37]. The criteria for acceptability were above .80.

Further deletions and modifications were made following additional comments from the panel regarding clarity, length, and duplication. The adjusted items were reviewed again by two professors of nursing.

### Scale evaluation

#### Phase 4: Main survey

##### Participants and settings

The main survey participants were school nurses in Korea. They were divided into five groups on the basis of the length of experience as a school nurse for the discriminative validity test: Novice Beginner (< 1 Year), Advanced Beginner (1–3 Years), Competent (3–6 Years), Proficient 1 (6–16 years), and Proficient 2 (≥16 years) [10].

A sample size of 500 was determined using five times the 92 items on the initial scale needed for the factor analysis [38] with an anticipated dropout rate of approximately 10%. The minimum sample size per group was 40, considering a Cohen’s medium effect size of .25 [39].

A total of 387 participants took part in the study. Although the total figure did not reach the targeted sample size, there were more than 300 cases for the factor analysis and more than 40 school nurses for each group. One respondent was ultimately excluded for giving the identical answer to all items.

##### Data collection

Data were collected from an online survey from November 26 to December 31, 2018, and consisted of 92 initial items, 30 items from the TCS [35], and 10 items related to the participants’ demographic characteristics. We contacted the Korea School Nurses Association to recruit the participants and asked a local representative of the association to send e-mails and text messages regarding participant recruitment to school nurses who were local members.

##### Data analysis

The data were analyzed using IBM SPSS 25.0 (Armonk, NY; IBM Corp.) and AMOS 25.0 (Chicago, IL; IBM SPSS Statistical Programs). Items with an absolute value of 2 or more for skewness and kurtosis were dropped [40]. Items-total correlations that were above .30 were regarded as acceptable.

The Kaiser–Meyer–Olkin (KMO) sample fit scale and Bartlett’s test of sphericity was performed with a KMO value ≥ .50 and a *p*-value for Bartlett’s test < .05 [38] to examine the appropriateness of the factor analysis.

Exploratory Factor Analysis (EFA) was conducted using principal axis factoring and oblique rotation using SPSS to select items that fit the factors in the research framework and to reduce the number of items. The criteria for deleting items were a cumulative value of .40 or less and a factor loading value of .30 or less [41]. Items were deleted until the number of extracted factors having eigenvalue =1 matched the factors of the hypothesized model based on the research framework.

The convergent validity and model fit of the scale was analyzed using Confirmatory Factor Analysis (CFA). The convergent validity of the items was verified through the following criteria: standardized factor loading (SFL ≥ 0.50), critical ratio (CR ≥ 1.96), *p*-value (*p* < .05), average variance extracted (AVE ≥ 0.50), construct reliability ≥ .70, and squared multiple correlation (SMC ≥ .40) [42, 43]. The criteria used for the fit indices included Normed χ^2^ (CMIN/*df)* ≤ 3, Comparative Fit Index (CFI) ≥ .90, Turker Lewis Index (TLI) ≥ .90, Root Mean Square Error of Approximation (RMSEA) ≤ .08, and Standardized Root Mean-square Residual (SRMR) ≤ .05 [43, 44].

The discriminative validity test was analyzed using the school nurse experience groups as these groups have been shown to have different levels of competency in previous studies [45, 46]. An analysis of covariance (ANCOVA) was conducted by assigning covariates to the variables that have confirmed differences using a chi-square test or one-way ANOVA.

The TCS [35] was modified for the school context and was used to assess criterion validity. Triage is one emergency nursing’s main features and a major challenge for school nurses [10]. The TCS [35] has several similarities to the sub-attributes of the competencies derived in this study. The relationship between the developed scale and the TCS were analyzed using Pearson’s correlation coefficients, with *r*s .40–.80 being regarded as acceptable [47].

The reliability of the whole scale and each factor was deemed to be acceptable if the Cronbach’s α > .65 [28].

### Final scale

#### Phase 5: final scale

The scale was finalized with items derived from the scale evaluation process. Five professors of nursing reviewed the final items to improve the items’ readability and comprehensibility.

## Results

### Scale development

#### Clarification of concept and development of the initial items

The school nurse’s emergency nursing care competencies and behavioral indicators were developed with 4 factors, 18 competencies, and 65 behavioral indicators. In addition, 20 major school emergencies were identified (Table 1) on the basis of the analysis of 23,985 accidents from School Safety Insurance Federation and literature on school emergencies [10, 48], Overall, 31 items were developed for the major assessments and interventions at the school.

**Table 1:** Classification of School Emergencies

Classification (*n* = 20)		Note
Injury		
Musculoskeletal	Fracture, Sprain, Strain, Dislocation^†^	Frequent Injury (1st)
Face	Eye injury	
	Dental & Oral	
	Nose	
Skin	Skin wound requiring suture	Frequent injury (2nd)
	Burn	
Head	Skull Fracture, Concussion, Scalp Damage	Serious Injury
Other	Choking, Abdominal Injury, Bites, Toxic Exposure, Genital Injury, Rape, Suicide	
Sudden Symptoms		
Neurological	Syncope, Unconsciousness^†^	Serious Symptoms
	Severe headache	
Cardiovascular and Respiratory	Dyspnea, Hyperventilation, Pneumothorax	Serious Symptoms
	Chest Pain, Arrhythmia, Tachycardia, Hypertension	Serious Symptoms
	Heart Arrest	
Gastrointestinal	Severe Abdominal Pain	
Other	Psychiatric Symptoms, Dehydration	
Deterioration of Disease		
Allergy	Allergic Reaction, Anaphylaxis^†^	Specified in Law
Cardiovascular and Respiratory	Asthma	
Endocrine	Hypoglycemia & Hyperglycemia	Specified in Law
Neurological	Seizures	Serious Symptoms
Other	Hemophilia	

*Note*: ^†^Included in the final version scale

The initial scale included 165 items: 21 on the *Professional, Ethical, and Legal Practice* factor, 94 on the *Care Provision* factor, 33 on the *Leadership and Management* factor, and 17 on the *Professional, Personal, and Quality Development* factor.

#### Content validity of initial items

The content validity analysis showed that one competency, nine behavioral indicators, and eight items had a CVI-I less than .80. All behavior indicators were dropped except for one that moved to another factor. Additional items were excluded or modified when they belonged to excluded competency and behavioral indicator, or demonstrated ambiguity or overlap (Fig. 1). Based on the similarity between factors, the *Professional, Ethical, and Legal Practice* factor was renamed as the *Ethical and Legal Practice* factor, and the *Professional, Personal, and Quality Development* factor was renamed as the *Professionalism and Quality Development* factor.

### Scale evaluation

#### Participant characteristics

A total of 386 participants were included in this study. Of whom, 162 (42.2%) participants had experience in caring for severe emergency patients at school, while 88 (22.8%) participants held certifications related to emergency nursing care such as Basic Life Support Provider, Advanced Life Support Instructor, First Aid Rescue, and First Aid Instructor (Table 2).

**Table 2:** General Demographic Characteristics of Participants (N = 386)

	Characteristics	Total (*N =* 386)		Group A (*n* = 75)		Group B (*n* = 50)		Group C (*n* = 56)		Group D (*n* = 105)		Group E (*n* = 100)	
		*n* (%) or *M* (*SD*)		*n* (%) or *M* (*SD*)		*n* (%) or *M* (*SD*)		*n* (%) or *M* (*SD*)		*n* (%) or *M* (*SD*)		*n* (%) or *M* (*SD*)	
Gender	Male	2	(0.5)	0	(0.0)	1	(2.0)	1	(1.8)	0	(0.0)	0	(0.0)
	Female	384	(99.5)	75	(100.0)	49	(98.0)	55	(98.2)	105	(100.0)	100	(100.0)
Age in years		41.09	(10.08)	31.65	(6.15)	32.94	(6.80)	36.54	(8.14)	43.49	(6.42)	52.22	(4.59)
Education	Doctoral Degree	4	(1.0)	0	(0.0)	0	(0.0)	0	(0.0)	0	(0.0)	4	(4.0)
	Master’s Degree	73	(18.9)	7	(9.3)	1	(2.0)	4	(7.1)	22	(21.0)	39	(39.0)
	Bachelor’s Degree	292	(75.7)	65	(86.7)	42	(84.0)	48	(85.7)	81	(77.1)	56	(56.0)
	Associate Degree	17	(4.4)	3	(4.0)	7	(14.0)	4	(7.1)	2	(1.9)	1	(1.0)
Workplace	Elementary School	210	(54.4)	49	(65.3)	28	(56.0)	22	(39.3)	46	(43.8)	65	(65.0)
	Middle School	105	(27.2)	20	(26.7)	14	(28.0)	21	(37.5)	33	(31.4)	17	(17.0)
	High School	71	(18.4)	6	(8.0)	8	(16.0)	13	(23.2)	26	(24.8)	18	(18.0)
Length of School Nurse Experience in years		10.28	(9.80)	0.88	(0.35)	2.32	(0.56)	4.16	(0.94)	10.37	(3.31)	24.66	(5.74)
Length of Hospital Nurse Experience in years		4.83	(4.38)	5.48	(5.69)	5.18	(4.45)	5.13	(3.80)	5.36	(4.55)	3.41	(2.82)
Emergency Experience	Yes	162	(42.2)	14	(18.7)	18	(36.0)	20	(35.7)	48	(45.7)	62	(62.0)
	No	224	(58.0)	61	(81.3)	32	(64.0)	36	(64.3)	57	(54.3)	38	(38.0)
Certificate Related to Emergency Nursing Care	Yes	88	(22.8)	15	(20.0)	4	(8.0)	13	(23.2)	30	(28.6)	26	(26.0)
	No	298	(77.2)	60	(80.0)	46	(92.0)	43	(76.8)	75	(71.4)	74	(74.0)

*Note*. *M,* mean; *SD,* standard deviation; Group A: Novice Group (< 1 year); Group B: Advanced Beginner Group (≥ 1 but < 3 years); Group C: Competent Group (≥ 3 but < 6 years); Group D: Proficient 1 Group (≤ 6 but < 16 years); Group E: Proficient 2 Group (≥ 16 years)

#### Items analysis

Four items on the *Ethical and Legal Practice* factor and one item on the *Care Provision* factor had an absolute value of skewness and kurtosis of more than 2. Further, three items showed lower item-total correlations than the criteria (*r* ≥ .30). Thus, eight items were dropped.

#### Validity analysis

##### Factor analysis

The KMO value was high (.97, *p* < .001) as was Bartlett’s test of sphericity, demonstrating the adequacy of the data for producing a reliable factor solution.

The number of items was reduced to 37 through sequential deletion, taking into consideration the factors of the research framework, the importance of the items, cumulative value, and the factor loading value. The total explanatory variance was 61.50%. Item 6 (*Ethical Practices)* had a cumulative value of .39, and Item 66 (*Delegation and Supervision)* had a factor loading < .30, but they were not dropped to maintain the construct of the factors.

The adjusted scale including 37 items was analyzed for its convergent validity and model fit. The fit indices did not meet criteria for an acceptable fit, and the modification indices indicated a high correlation between the *Care Provision* factor and the *Leadership and Management* factor. The *Care Provision* and the *Leadership and Management* factors were integrated into a single factor—*Emergency Care Provision and Management*. Sub-factors were created by separating *Assessment, Diagnosis, and Planning* and *Intervention, Evaluation, Therapeutic Communication, and Relationships* because they tended to load differently. Item 41, initially dropped for failing to meet the validity criteria, was added back to the final scale, as it was a characteristic intervention used in schools. Eight items (22, 24, 25, 37, 76, 78, 75, and 80) were dropped sequentially as the modification indices were high, and the items were considered less important. The final version of the School Nurse’s Emergency Nursing Care Competency Scale (ENCCS_SN) consisted of the 30 remaining items (Fig. 1).

The fit indices of the final scale met criteria for acceptable model fit (Table 3). The convergent validity analysis of each of the factors in the final scale had AVE values ranging from .57 to .95 and construct reliability values ranging from .79 to .98, meeting their criteria. The SFL for item 41, the reverse item, was .34, and the SMC = .12, which did not meet the criteria. However, the SFL of the other items ranged from .62 to .86 and the SMC from .39 to .74. The convergent validity analysis of the sub-factors for the *Emergency Care Provision and Management* factor showed an AVE of .95 and construct reliability of .98, meeting the criteria. In addition, the SFL of the sub-factors ranged from .88 to .99 and the SMC from .78 to .98, also meeting criteria (Supplementary Material 1).

**Table 3:** Model Fit (N = 386)

Model (No. of items)	CMIN/*df*	CFI	TLI	RMSEA	SRMR
	≤ 3	≥ .90	≥ .90	≤ .08	≤ .05
Initial (37)	3.88	.85	.84	.07	.06
Final (30)	2.67	.92	.91	.07	.05

*Note*. *CMIN/df: Normed χ*^*2*^*; CFI: Comparative Fit Index; TLI: Tucker Lewis Index; RMSEA: Root Mean Square Error of Approximation; SRMR: Standardized Root Mean-square Residual*

##### Discriminative validity

Having emergency experience (χ^2^ = 35.43, *df = 4, p* < .001) and hospital nurse experience (*F* = 4.06, *df = 3, p* = .007) were assigned as covariates. Table 4 shows the results of the differences between the school nurse experience groups (*F* = 3.64, *df = 4, p* = .006). The competencies of the short-experience groups were lower than those of the long-experience groups.

**Table 4:** Differences in the Emergency Nursing Care Competency by School Nurse Experience (*N* = 386)

Classification	*n*	*M* (*SD)*		*F*	*p*	
Group A	75	84.28	(16.73)	3.64	.006	A, B, C < D, E
Group B	50	86.58	(18.23)			
Group C	56	85.84	(19.93)			
Group D	105	93.56	(17.21)			
Group E	100	93.32	(18.94)			

*Note*: Adjusted by emergency experience; hospital nurse experience. Post hoc analysis using LSD; *M,* mean; *SD,* standard deviation; Group A: Novice Group (< 1 year); Group B: Advanced Beginner Group (≥ 1 but < 3 years); Group C: Competent Group (≥ 3 but < 6 years); Group D: Proficient 1 Group (≤ 6 but < 16 years); Group E: Proficient 2 Group (≥ 16 years)

##### Criterion validity

Correlation analyses of the scores measured by the TCS (Moon & Park, 2018) showed the overall correlation coefficient was *r =* .86 (*p* < .001), with *r =* .45 (*p* < .001) for the *Ethical and Legal Practice* factor, *r =* .87 (*p* < .001) for the *Emergency Care Provision and Management* factor, and *r =* .55 (*p* < .001) for the *Professionalism and Quality Development* factor.

#### Reliability: internal consistency

Analyses of the reliability of the final scale found no items increased the Cronbach’s α of a factor when deleting items. The Cronbach’s α for the whole scale was .96. For each factor, it ranged from .74 to .96.

### The final scale

The final scale consisted of 30 items loading on three factors: The *Ethical and Legal Practice* factor, the *Emergency Care Provision and Management* factor, and the *Professionalism and Quality Development* factor. The sub-factors under the *Emergency Care Provision and Managemen*t factor were the *Clinical Decision-Making* sub-factor, the *Care Provision* sub-factor*,* and the *Leadership and Management* sub-factor. The final scale included 22 behavioral indicators of competency (Table 5). One reverse-scored item was changed to a positive item in the final scale. The final scale used a self-report format with participants responding on a 5-point rating scale, ranging from 0 to 4 points for convenience of interpretation (0 = *never*, 1 = *rarely*, 2 = *sometimes*, 3 = *often,* and 4 = *always*). Higher scores indicated higher ENCC (Supplementary Material 2). The mean score was 89.67 (*SD =* 18.46).

**Table 5:** Behavior Indicators of Competency in the Final Scale

Domain			Behavior Indicator of Competency	Item No.	
				First Version	Final Version
**F1**		Ethical Practice	Maintains the confidentiality of the patient when delivering emergency nursing care.	6, 7	1, 2
		Legal Practice	Practices in accordance with related laws and regulations.	8	3
**F2**	F2–1	Assessment & Diagnosis	Collects subjective and objective data of patients promptly and systematically.	20, 21, 23, 18, 14	4, 5, 6, 7, 8
			Analyzes the collected data comprehensively, considering the characteristics of school emergencies.	28	9
			Prioritizes promptly based on evidence.	34	10
			Promptly diagnoses health problems based on evidence.	32	11
		Planning	Promptly establishes an emergency nursing care plan considering school resources.	15	12
	F2–2	Intervention	Promptly provides emergency nursing care interventions using school resources.	51, 38, 41, 43	13, 14, 15, 16
		Evaluation	Evaluates the progress of emergency nursing care.	59	17
			Evaluates the outcome of emergency nursing care.	63	18
		Therapeutic Communication & Relationships	Provides accurate detailed information to patients and parents.	69	19
			Documents the emergency nursing intervention and the patient’s response.	62	20
			Interacts in a manner that is respectful to patients and parents.	77	21
	F2–3	Safe Environment	Prepares and maintains emergency supplies in accordance with regulations.	74	22
			Identifies and prepares for emergencies at school.	79	23
			Establishes a school emergency system.	81	24
		Delegation & Supervision	Establishes a delegation system for emergency nursing care in accordance with regulations.	66	25
		Inter-professional	Reasonably resolves conflicts related to school emergencies.	85	26
		Health Care	Makes an effort to enhance the staff’s emergency response abilities.	86	27
**F3**		Enhancement of the profession	Continuously engages in education to enhance the profession.	91	28
		Quality Improvement	Participates in evidence-based research to improve emergency nursing care practice.	92	29
			Identifies and improves problems in practice.	90	30

*Note*: F1: Ethical and Legal Practice; F2: Emergency Care Provision and Management; F2–1: Clinical Decision Making (Assessment, Diagnosis, Plan); F2–2: Care Provision (Intervention, Evaluation, Therapeutic Communication & Relationships); F2–3: Leadership and Management; F3: Professionalism and Quality Development

## Discussion

This study developed the first self-report scale designed to measure school nurses’ ENCC. The Emergency Nursing Care Competency Scale for School Nurses (ENCCS_SN) demonstrated good reliability and met criteria for content and construct validity. Additionally, the ENCCS_SN represents the first scale to incorporate items pertaining to the characteristics and contents of school emergency nursing care. The validity of the scale was determined by identifying the constructs of the concepts and developing items that effectively measured each construct [11, 28]. In this study, the NCCCF [29] was used as a theoretical framework to clarify the scale’s composition. It is particularly meaningful that the ENCCS_SN was consistent with the nursing competencies, factors, and behaviors identified in the NCCCF [29]. Since nursing competency is the ability to perform common and basic nursing tasks [49], these results suggest that overall nursing competencies remain the same across areas of specialization and practice.

However, the sub-factors of the *Emergency Care Provision and Management* factor in the ENCCS_SN were constructed differently from the NCCCF [29] since the item means related to assessment and diagnosis on the factor analysis were lower than the other items. This finding suggests that assessment and diagnosis is the most difficult part of emergency nursing care [50], and school nurses also find it challenging to assess emergency patients in the schools because schools have limited medical resources [10]. Therefore, developing and providing an educational program or support strategies for school nurses that focus on assessment and diagnosis may need to be prioritized.

Item 41 (*Hospital Referral and Transfer*) from the intervention items had an SFL of .34 and an SMC of .12, which were lower than the criteria. However, an SFL of .34 was deemed acceptable by Tabachnick and Fidell [51]. The low convergent validity of Item 41 was due to its lower mean compared to the other intervention items, potentially owing to school nurses’ challenges with transferring patients [10]. Item 41 may also have been developed as a reverse-scored item, confusing respondents. Other studies have reported that reverse-scored items tend to have low consistency [28]. Thus, Item 41 was revised to be a positive item in the final scale to avoid confusion with the reverse wording. In future studies, Item 41 should be evaluated.

In this study, the discriminative validity by experience group was confirmed, which showed the usefulness of the scale in explaining the relationship between experience and competency. The ENCC of the group with more than 6 years of experience was higher than other groups, whereas previous research on emergency room nurses’ ENCC reported that the ENCC of those with more than 2 years’ experience was higher than other groups [52]. One reason that school nurses take longer than hospital nurses to increase their competency could be that university curricula have not yet fully address school emergency nursing care competencies that require extensive knowledge in the context of the schools’ limited medical resources [53]. In addition, insufficient on-site support for improving the nurses’ ENCC has been reported [54, 55]. Given most school nurses work alone, developing a competency-based education program [56] that can improve the school nurses’ ENCC at the beginning of their experience is necessary.

### Limitations

In this study, the same sample was used for both the EFA and CFA. Because of the number of items and factors developed in the study, it was difficult to obtain sufficiently large samples that could be divided. In this study, EFA was applied as a process for refining scales with the limitation that CFA would be repeating relationships established through the EFA [57].

The TCS [35] used to assess criterion validity in this study is not the gold standard. It has been developed for emergency room nurses, not school nurses. Nevertheless, the TCS [35] was used because, to date, there is no gold standard for emergency nursing care, and triage is an integral component of emergency nursing care.

## Conclusions

The results demonstrated that the ENCCS_SN is a valid and reliable scale for measuring school nurses’ ENCC. The scale can be useful to assess school nurses’ ENCC and develop a competency-based education program and nursing curriculum for school nurses. Furthermore, the ENCCS_SN could help identify related factors in developing effective interventions or policies as well as evaluate the policy outcomes or interventions related to school emergency nursing care.

It is acknowledged that the cut-off value in this study could not be presented due to the lack of gold standard in the ENCC. Therefore, further research should be considered in order to identify what variables can be used to measure the outcomes of school nurses’ ENCC or to develop a scale used to measure them. In this study, the NCCCF [29] was applied as the research framework to develop a scale that can be used internationally even though each item was developed in the context of Korean school emergency nursing care. The scale was developed in Korean and then translated into English, but the validity of the translated scale was not verified. It should be further noted that, given the international differences in school nursing practices, the scale should be used once the scale’s validity has been verified following country-specific modification for school nursing practice.

## Supplementary Information

**Additional file 1 Supplementary material 1:** Result of the Confirmatory Factor Analysis. **Supplementary material 2:** The Emergency Nursing Care Competency Scale for School Nurse: ENCCS_SN.

## Data Availability

All data generated or analysed during this study are included in this published article.

## Abbreviations

ANCOVA: Analysis of Covariance
AVE: Average Variance Extracted
CFI: Comparative Fit Index
CFA: Confirmatory Factor Analysis
CVI-I: Content Validity Index for Item
CR: Critical Ratio
ENCC: Emergency Nursing Care Competency
ENCCS_SN: School Nurse’s Emergency Nursing Care Competency Scale
EFA: Exploratory Factor Analysis
ICN: International Council of Nurses
KMO: Kaiser–Meyer–Olkin
NCCCF: Nursing Care Continuum Competencies Framework
RMSEA: Root Mean Square Error of Approximation
SMC: Squared Multiple Correlation
SFL: Standardized Factor Loading
SRMR: Standardized Root Mean-square Residual
TCS: Triage Competency Scale
TLI: Turker Lewis Index

## References

[1] Elgie R, Sapien R, Fullerton L, Moore B (2010). School nurse online emergency preparedness training: an analysis of knowledge, skills, and confidence. J Sch Nurs.

[2] Mobarak AS, Afifi RM, Qulali A (2015). First aid knowledge and attitude of secondary school students in Saudi Arabia. Health.

[3] Smith CM, Colquhoun MC (2015). Out-of-hospital cardiac arrest in schools: a systematic review. Resuscitation..

[4] Kiyohara K, Sado J, Kitamura T, Ayusawa M, Nitta M, Iwami T, Nakata K, Sato Y, Kojimahara N, Yamaguchi N, Sobue T, Kitamura Y, for the SPIRITS Investigators (2018). Epidemiology of pediatric out-of-hospital cardiac arrest at school—an investigation of a nationwide registry in Japan. Circ J.

[5] School Safety Mutual Aid Association (2019). School accident statistics. School Safety and Insurance Federation, Seoul, South Korea.

[6] Special Education Policy Division (2019). Special education statistics. Ministry of Education, Student Services Bureau, Daejon, South Korea.

[7] Evans W, Ficca M (2012). The school nurse role in preparing for sudden cardiac arrest in the school setting. J Sch Nurs.

[8] Murphy MK (2014). Emergency anaphylaxis at school. Am J Nurs.

[9] Schoessler S, White MV (2013). Recognition and treatment of anaphylaxis in the school setting: the essential role of the school nurse. J Sch Nurs.

[10] Yoon J, Lee I (2017). The emergency care experience and demand for support of school nurse. J Korean Acad Community Health Nurs.

[11] Lee R (2011). The role of school nurses in delivering accessible health services for primary and secondary school students in Hong Kong: the role of school nurses in Hong Kong. J Clin Nurs.

[12] Lineberry M, Whitney E, Noland M (2018). The role of school nurses, challenges, and reactions to delegation legislation: a qualitative approach. J Sch Nurs.

[13] Boudreaux S, Broussard L (2012). Sudden cardiac arrest in schools: the role of the school nurse in AED program implementation. Issues Compr Pediatr Nurs.

[14] Nilsson J, Johansson S, Nordström G, Wilde-Larsson B (2020). Development and validation of the ambulance nurse competence scale. J Emerg Nurs.

[15] Wilkinson CA (2013). Competency assessment tools for registered nurses: an integrative review. J Contin Educ Nurs.

[16] Jeon Y, Meretoja R, Vahlberg T, Leino-Kilpi H (2020). Developing and psychometric testing of the anaesthesia nursing competence scale. J Eval Clin Pract.

[17] Resha C (2009). School nurse competencies. How can they assist to ensure high-quality care in the school setting?. NASN Sch Nurse.

[18] Dellai M, Mortari L, Meretoja R (2009). Self-assessment of nursing competencies–validation of the Finnish NCS instrument with Italian nurses. Scand J Caring Sci.

[19] Juntasopeepun P, Turale S, Kawabata H, Thientong H, Uesugi Y, Matsuo H (2019). Psychometric evaluation of the nurse competence scale: a cross-sectional study. Nurs Health Sci.

[20] Gunawan J, Aungsuroch Y, Fisher ML, Marzilli C, Liu Y (2020). Factors related to the clinical competence of registered nurses: systematic review and meta-analysis. J Nurs Scholarsh.

[21] Hsieh PL, Chen CM (2017). Long term care nursing competence and related factors among Taiwanese nurses: a national survey for those who completed the LTC training course. Geriatr Nurs.

[22] Yamamoto Y, Okuda R, Fukada M (2021). Factors affecting clinical nursing competency: a cross sectional study. Yonago Acta Med.

[23] Delaney MM, Friedman MI, Dolansky MA, Fitzpatrick JJ (2015). Impact of a sepsis educational program on nurse competence. J Contin Educ Nurs.

[24] Mirbagher Ajorpaz N, Zagheri Tafreshi M, Mohtashami J, Zayeri F, Rahemi Z (2016). The effect of mentoring on clinical perioperative competence in operating room nursing students. J Clin Nurs.

[25] Marin SM, Hutton A, Witt RR (2020). Development and psychometric testing of a tool measuring nurses’ competence for disaster response. J Emerg Nurs.

[26] Connecticut State Department of Education (2014). Competency in school nurse practice, 2nd ed. Connecticut State Department of Education, Connecticut’s Official State Website.

[27] Park K, Bea E (2012). A delphi study of developing competency model for Korean health teachers. J Korean Soc School Health.

[28] DeVellis RF (2017). Scale development: theory and applications.

[29] International Council of Nurses (2008). Nursing care continuum framework and competencies 2008*.* ICN Regulation Series, Geneva, Switzerland, 2008.

[30] Elo S, Kyngas H (2008). The qualitative content analysis process. J Adv Nurs.

[31] Abdolrahimi M, Ghiyasvandian S, Zakerimoghadam M, Ebadi A (2017). Therapeutic communication in nursing students: a Walker & Avant concept analysis. Electron Physician.

[32] Chivima B (2014). Ethical practice. Nurs Stand.

[33] Yun S, Lee J (2014). Concept analysis to therapeutic communication of nursing students. J Korean Acad Community Health Nurs.

[34] Dunn SV, Lawson D, Robertson S, Underwood M, Clark R, Valentine T, Walker N, Wilson-Row C, Crowder K, Herewane D (2000). The development of competency standards for specialist critical care nurses. J Adv Nurs.

[35] Moon S, Park Y (2018). Development of the triage competency scale for emergency nurses. J Korean Acad Nurs.

[36] Wihlborg J, Edgren G, Johansson A, Sivberg B (2014). The desired competence of the Swedish ambulance nurse according to the professionals—a Delphi study. Int Emerg Nurs.

[37] Zamanzadeh V, Ghahramanian A, Rassouli M, Abbaszadeh A, Alavi-Majd H, Nikanfar AR (2015). Design and implementation content validity study: development of an instrument for measuring patient-centered communication. J Caring Sci.

[38] Kang H (2013). A guide on the use of factor analysis in the assessment of construct validity. J Korean Soc Nurs Sci.

[39] Cohen J (2013). Statistical power analysis for the behavioral sciences. Routledge.

[40] Bea J (2012). Health and medical statistics of doctor Bae in picture.

[41] Kim C (2016). Misuse of exploratory factor analysis and its remedies. Korean Assoc Survey Res.

[42] Song J (2013). SPSS/Amos statistical analysis method. 4th ed. Paju, South Korea: 21st Century Book.

[43] Woo J (2017). Concept and understanding of structural equation model.

[44] Bae B (2017). Amos 24 structural equation modeling.

[45] Roh YS, Issenberg SB, Chung HS, Kim SS, Lim TH (2013). A survey of nurses’ perceived competence and educational needs in performing resuscitation. J Contin Educ Nurs.

[46] Salonen AH, Kaunonen M, Meretoja R, Tarkka MT (2007). Competence profiles of recently registered nurses working in intensive and emergency settings. J Nurs Manag.

[47] Lee E, Lim N, Park H, Lee I, Kim J, Bae J (2009). Nursing research and statistical analysis.

[48] Amanullah S, Heneghan JA, Steele DW, Mello MJ, Linakis JG (2014). Emergency department visits resulting from intentional injury in and out of school. Pediatrics..

[49] Emergency Nurses Association (2011). Competencies for clinical nurse specialists in emergency care.

[50] Choi U, Cho K (2008). The study of needs and demands for first aid education of school health educator. J Korean Soc Emerge Med Technol.

[51] Tabachnick BG, Fidell LS (2013). Using multivariate statistics: Pearson new international edition. Volume 6.

[52] McCarthy G, Cornally N, Mahoney CO, White G, Weathers E (2013). Emergency nurses: procedures performed and competence in practice. Int Emerg Nurs..

[53] Newell ME (2013). Patients of the future: a survey of school nurse competencies with implications for nurse executives in the acute care settings. Nurs Adm Q.

[54] Kruger BJ, Radjenovic D, Toker KH, Comeaux JM (2009). School nurses who only care for children with special needs: working in a teacher’s world. J Sch Nurs.

[55] Lee J, Lee B (2014). Role adaptation process of elementary school health teachers: establishing their own positions. J Korean Soc Nurs Sci.

[56] Hoseini SD, Khankeh HR, Dalvandi A, Saberinia A, Rezasoltani P, Mirzaeirad SZ (2018). Comparing the effect of the two educational methods: competency-based, and lecture, on the knowledge and performance of nurses in the field of hospital triage. Health Emerge Dis Quarterly.

[57] Knekta E, Runyon C, Eddy S (2019). One size doesn’t fit all: using factor analysis to gather validity evidence when using surveys in your research. CBE Life Sci Educ.

