# Supplementary material for: Development of emergency nursing care competency scale for school nurses
Source: BMC Nurs. 2021 Apr 14;20:63. doi: 10.1186/s12912-021-00580-9 (PMC8045335; doi:10.1186/s12912-021-00580-9)
Supplement: Supplementary file 1 — Additional file 1 Supplementary material 1: Result of the Confirmatory Factor Analysis. Supplementary material 2: The Emergency Nursing Care Competency Scale for School Nurse: ENCCS_SN. [file 12912_2021_580_MOESM1_ESM.docx]

**Development of** **Emergency Nursing Care Competency Scale for School Nurses**

**Jaehee Yoon**

**Supplementary material 1:** Result of the Confirmatory Factor Analysis

| Domain | | Item no. | Item | Factor Loading | CR  ≥1.96 | *p*  value | SFL  ≥ .50 | SMC  ≥ .40 | AVE  ≥ .50 | Construct Reliability ≥ 70 |
| --- | --- | --- | --- | --- | --- | --- | --- | --- | --- | --- |
| F1 | |  |  |  |  |  |  |  | .57 | .79 |
|  | Ethical Practice | 6 | I do not break the confidentiality of the patients acquired while delivering emergency nursing care. | 1 |  |  | .65 | .42 |  |  |
|  |  | 7 | I keep my emergency nursing care records in a secure location. | 1.60 | 10.51 | <.001 | .66 | .44 |  |  |
|  | Legal Practice | 8 | I adhere to laws and regulations related to the school emergency nursing care. | 1.51 | 11.61 | <.001 | .84 | .71 |  |  |
| F2 | | | |  |  |  |  |  | .95 | .98 |
|  | F2-1 | | | 1 |  |  | .88 | .78 | .71 | .96 |
|  | Assessment & Diagnosis | 20 | I can promptly and systematically assess the state of consciousness, the abnormal appearance, and accidents of patients with head injuries. | 1.05 | 21.35 | <.001 | .84 | .71 |  |  |
|  |  | 21 | I can promptly and systematically assess the mental status, neurological conditions, medical history, and so on, in patients with syncope or who are conscious. | 1.06 | 21.49 | <.001 | .84 | .71 |  |  |
|  |  | 23 | I can promptly and systematically assess the breathing, skin conditions, and medical history of patients with allergies. | 1 |  | <.001 | .85 | .73 |  |  |
|  |  | 18 | I can promptly and systematically assess the degree of injury (asymmetry, range of motion, swelling, ecchymosis, neurovascular damage, etc.) in patients with musculoskeletal injuries. | 1.00 | 19.40 | <.001 | .79 | .63 |  |  |
|  |  | 14 | I can reassess patient at regular intervals and reassign triage. | 0.86 | 17.10 | <.001 | .73 | .54 |  |  |
|  |  | 28 | I can comprehensively analyse collected data, considering the school emergency patients’ characteristics. | 0.99 | 22.22 | <.001 | .86 | .74 |  |  |
|  |  | 34 | I can promptly determine the necessary hospital referral, transfer, and observation level of emergency patients based on evidence. | 0.89 | 18.55 | <.001 | .77 | .60 |  |  |
|  |  | 32 | I can promptly assign appropriate diagnoses based on evidence. | 0.96 | 20.15 | <.001 | .81 | .66 |  |  |
|  | Planning | 15 | I can establish an emergency nursing care plan based on school and family resources. | 0.85 | 15.70 | <.001 | .69 | .48 |  |  |
|  | F2-2 | | | 0.81 | 12.46 | <.001 | .99 | .98 | .57 | .92 |
|  | Intervention | 51 | I can dress the affected patients wounds promptly and accurately. | 1.16 | 11.90 | <.001 | .71 | .51 |  |  |
|  |  | 38 | I can promptly report an emergency situation. | 1.21 | 12.73 | <.001 | .78 | .61 |  |  |
|  |  | 41^†^ | I find it difficult to coordinate emergency patient transfers and refer them to the hospital. | 0.62 | 6.32 | <.001 | .34 | .12 |  |  |
|  |  | 43 | I can do CPR accurately and promptly. | 1.31 | 12.27 | <.001 | .74 | .54 |  |  |
|  | Evaluation | 59 | I check if there are any problems or deficiencies in the emergency nursing process. | 1.07 | 11.65 | <.001 | .69 | .48 |  |  |
|  |  | 63 | I can evaluate the nursing diagnosis and implementation by checking the medical results of the emergency patient. | 1.32 | 13.01 | <.001 | .80 | .64 |  |  |
|  | Therapeutic Communication & Relationships | 69 | I provide patients (or caregivers) with detailed information such as emergency patient’s condition, follow-up care, and symptoms of deterioration. | 1.25 | 12.42 | <.001 | .75 | .57 |  |  |
|  |  | 62 | I can accurately and precisely document emergency nursing interventions and a patient’s conditions. | 1.34 | 12.92 | <.001 | .80 | .63 |  |  |
|  |  | 77 | I understand the negative reactions of emergency patients or parents. | 1 |  | <.001 | .62 | .39 |  |  |
|  | F2-3 | | | 0.95 | 14.98 | <.001 | .96 | .92 | .60 | .90 |
|  | Safe  Environment | 74 | I prepare and maintain emergency supplies to be ready for an emergency. | 1 |  | <.001 | .75 | .56 |  |  |
|  |  | 79 | I am prepared for emergencies that may occur to children with health problems. | 1.01 | 16.65 | <.001 | .82 | .68 |  |  |
|  |  | 81 | I have established a specific school emergency system that includes patient transfer and reporting, emergency resources, and division of roles and responsibilities. | 1.01 | 14.92 | <.001 | .75 | .56 |  |  |
|  | Delegation | 66 | I have established a clear emergency nursing care delegation system in the absence of a school nurse. | 1.16 | 13.82 | <.001 | .70 | .48 |  |  |
|  | Inter-professional Health Care | 85 | I try to reasonably resolve conflictual situations related to school emergencies. | 1.08 | 16.35 | <.001 | .81 | .66 |  |  |
|  |  | 86 | I regularly provide practical first aid training to staff. | 1.16 | 13.22 | <.001 | .67 | .45 |  |  |
| F3 | | | |  |  |  |  |  | .95 | .98 |
|  | Enhancement of the profession | 91 | I continuously acquire knowledge through books, research and training related to emergency nursing care | 1 |  |  | .84 | .70 |  |  |
|  | Quality Improvement | 92 | I actively cooperate with or participate in research to improve emergency nursing care practice. | 1.05 | 16.97 | <.001 | .80 | .63 |  |  |
|  |  | 90 | I apply and evaluate the latest developments or improvements introduced at seminars, training, and conferences on emergency nursing. | 1.14 | 17.81 | <.001 | .84 | .70 |  |  |

*Note*. ^†^Reverse item.; F1: Ethical and Legal Practice; F2: Emergency Care Provision and Management; F2-1: Clinical Decision Making (Assessment, Diagnosis, Plan); F2-2: Care Provision (Intervention, Evaluation, Therapeutic Communication & Relationships); F2-3: Leadership and Management; F3: Professionalism and Quality Development; CR, critical ratio; SFL, standardized factor loading; SMC, squared multiple correlation; AVE, average variance extracted

**Supplementary material 2:** The Emergency Nursing Care Competency Scale for School Nurse: ENCCS_SN

| Purpose | To measure the emergency nursing care competency of a school nurse |
| --- | --- |
| Method | The ICN Nursing Care Continuum Competencies Framework (ICN, 2008) was used as a research framework, and the major school emergency nursing care contents were identified and developed as items. |
| Type | Self-reported 5-point Likert scale ('0=never, '1=rarely', '2=sometimes', '3=often' and '4=always')  The total score ranges: 0∼120 |
| Target | School nurse |
| Composition | Total 30 items   \| Factor (the number of items) \| \| \| Item No. \| \| --- \| --- \| --- \| --- \| \| Ethical and legal practice (3) \| \| Ethical Practice (2) \| 1∼2 \| \| Legal Practice (1) \| 3 \| \| Emergency Care Provision and Management’  (24) \| Clinical Decision Making (9) \| Assessment & Diagnosis (8) \| 4∼11 \| \| Planning (1) \| 12 \| \| Care Provision (9) \| Intervention (4) \| 13∼16 \| \| Evaluation (2) \| 17∼18 \| \| Therapeutic Communication & Relationships (3) \| 19∼21 \| \| Leadership and Management (6) \| Safe Environment (3) \| 22∼24 \| \| Delegation (1) \| 25 \| \| inter-professional Health Care (2) \| 26∼27 \| \| Professionalism and Quality Development (3) \| \| Enhancement of the profession (1) \| 28 \| \| Quality Improvement (2) \| 29∼30 \| |
| Validity | - Content validity of initial items (Expert panel review): Consists of items of CVI .80 or higher - Convergent validity: convergent validity of three factors was confirmed (AVE=.57∼.95, CR=.79∼.98) - Discriminative validity: Significant mean difference between experience groups (F=4.06, 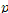=.007) - Criterion validity: The whole correlation coefficient with the criterion is .86, and the correlation coefficient between factors is .45 ~ .87 |
| Reliability | The whole Cronbach's α value is .96, and each factor Cronbach's α value is .74~.96 |
| Interpretation | Higher scores indicate higher emergency nursing care competency. |

The following items examine the school nurse's emergency nursing care competency for health-related school emergency patients. Read the items and mark ‘∨’ at the ones that you think best represent your thoughts or actions.

| No. | Items | never | rarely | sometimes | often | always |
| --- | --- | --- | --- | --- | --- | --- |
|  |  | (0) | (1) | (2) | (3) | (4) |
| 1 | I do not break the confidentiality of patients acquired during delivering emergency nursing care. |  |  |  |  |  |
| 2 | I keep my emergency nursing care records in a secure location. |  |  |  |  |  |
| 3 | I adhere to laws and regulations related to the school emergency nursing care. |  |  |  |  |  |
| 4 | I can promptly and systematically assess the state of consciousness, appearance abnormalities, and accidents of patients with head injuries. |  |  |  |  |  |
| 5 | I can promptly and systematically assess the mental status, neurological conditions, medical history, and so on, in patients with syncope or who are conscious. |  |  |  |  |  |
| 6 | I can promptly and systematically assess the breathing, skin conditions and medical history of patients with allergies. |  |  |  |  |  |
| 7 | I can promptly and systematically assess the degree of injury (asymmetry, range of motion, swelling, ecchymosis, neurovascular damage, etc.) in patients with musculoskeletal injuries. |  |  |  |  |  |
| 8 | I can reassess the patient at regular intervals and reassign triage. |  |  |  |  |  |
| 9 | I can comprehensively analyze collected data, considering school emergency patients’ characteristics. |  |  |  |  |  |
| 10 | I can promptly determine the hospital referral, transfer, and observation level of emergency patients based on evidence. |  |  |  |  |  |
| 11 | I can promptly assign appropriate diagnoses based on evidence. |  |  |  |  |  |
| 12 | I can establish an emergency nursing care plan based on school and family resources. |  |  |  |  |  |
| 13 | I can dress the affected patient's wounds promptly and accurately. |  |  |  |  |  |
| 14 | I can promptly report an emergency situation. |  |  |  |  |  |
| 15 | I find it difficult to coordinate emergency patient transfers and refer them to the hospital. |  |  |  |  |  |
| 16 | I can do CPR accurately and promptly. |  |  |  |  |  |
| 17 | I check if there are any problems or deficiencies in the emergency nursing process. |  |  |  |  |  |
| 18 | I can evaluate the nursing diagnosis and implementation by checking the medical results of the emergency patient. |  |  |  |  |  |
| 19 | I provide patients (or care givers) with detailed information such as emergency patient’s condition, follow-up care, and symptoms of deterioration. |  |  |  |  |  |
| 20 | I can accurately and precisely document emergency nursing interventions and a patient’s conditions. |  |  |  |  |  |
| 21 | I understand the negative reactions of emergency patients or parents. |  |  |  |  |  |
| 22 | I prepare and maintain emergency supplies to be ready for an emergency. |  |  |  |  |  |
| 23 | I am prepared for emergencies that may occur to children with health problems. |  |  |  |  |  |
| 24 | I have established a specific school emergency system that includes patient transfer and reporting, emergency resources, and division of roles and responsibilities. |  |  |  |  |  |
| 25 | I have established a clear emergency nursing care delegation system in the absence of a school nurse. |  |  |  |  |  |
| 26 | I try to reasonably resolve conflict situations related to school emergencies. |  |  |  |  |  |
| 27 | I regularly provide practical first aid training to staff. |  |  |  |  |  |
| 28 | I continuously acquire knowledge through books, research and training related to emergency nursing care |  |  |  |  |  |
| 29 | I actively cooperate with or participate in research to improve emergency nursing care practice. |  |  |  |  |  |
| 30 | I apply and evaluate the latest developments or improvements introduced at seminars, training, and conferences on emergency nursing. |  |  |  |  |  |
